# Nucleic acid-sensing-related gene signature in predicting prognosis and treatment efficiency of small cell lung cancer patients

**DOI:** 10.3389/fonc.2024.1394286

**Published:** 2024-04-12

**Authors:** Qianshi Liu, Zhaoshen Li, Na Li, Junjie Liu, Hong Wu, Jie Chen

**Affiliations:** ^1^ Department of Hepatobiliary Surgery, Affiliated Cancer Hospital of Guangxi Medical University, Nanning, China; ^2^ Department of Oncology and Cancer Institute, Sichuan Academy of Medical Sciences, Sichuan Provincial People’s Hospital, University of Electronic Science and Technology of China, Chengdu, China; ^3^ Shenzhen Engineering Center for Translational Medicine of Precision Cancer Immunodiagnosis and Therapy, YuceBio Technology Co., Ltd, Shenzhen, China

**Keywords:** small cell lung cancer, nucleic acid sensing-related genes, prognostic signature, tumor microenvironment, multiplex immunohistochemistry

## Abstract

**Introduction:**

Nucleic acid-sensing (NAS) pathways could induce innate and adaptive immune responses. However, rare evidence exhibited how the core genes of the NAS pathways affected the immune response and prognosis of small cell lung cancer (SCLC) patients.

**Methods:**

We conducted a comprehensive bioinformatic analysis based on the RNA profiles of 114 SCLC patients, including 79 from cBioPortal, 21 from GSE30219, and 14 from our sequencing data. The multiplex immunohistochemistry (mIHC) was used to characterize the role of NAS related genes in the tumor microenvironment (TME) of SCLC.

**Results:**

A prognostic model (7NAS risk model) was constructed based on 7 NAS-related genes which was demonstrated as an independent prognostic index. The low-risk group was identified to have a better prognosis and an immune-activated microenvironment in both the public datasets and our dataset. Intriguingly, mIHC data showed that CD45^+^ immune cells, CD8^+^ T lymphocytes, and CD68^+^ macrophages were prevalently enriched in low-risk SCLC patients and positively correlated with IRF1 expression. Additionally, Patients in the low-risk group might have superior responses to chemotherapy and immunotherapy.

**Conclusion:**

Conclusively, this study created a new risk model based on genes associated with NAS pathways which could predict the prognosis and response of treatment in patients with SCLC.

## Introduction

Small cell lung cancer (SCLC), a highly aggressive neuroendocrine malignancy, comprises about 15% of bronchogenic carcinomas (1–3). It is known for its swift progression, propensity for widespread metastasis, and overall dismal prognosis (4, 5). Platinum-based chemotherapy combined with etoposide remains the cornerstone of SCLC treatment, delivering an initially favorable response in patients (6, 7). Nonetheless, recurrence rates for SCLC are notoriously high. Recent strides in immunotherapy have opened promising avenues for treatment (1, 3, 7). However, only an estimated 30% of patients with SCLC experience benefits from such immunotherapeutic approaches (8). Currently, there is a lack of reliable biomarkers that can accurately predict the survival of patients and which patients will positively respond to chemotherapy or immunotherapy. Hence, there is a pressing need to identify novel molecular markers that can prognosticate survival and potentially guide therapeutic responses in SCLC patients.

The essential roles of nucleic acid-sensing (NAS) pathways in detecting microbial pathogens, through recognizing their nucleic acids and activating innate immunity, have been well-established (9), and their involvement in malignant tumors is now garnering increased attention (9, 10). Recent evidence indicates that NAS regulates DNA damage responses and monitors micronuclei, potentially impacting the treatment of malignant tumors (11). The primary role of NAS pathways is attributed to activating NAS, which can trigger an anti-tumor immune response (12). Radiotherapy and chemotherapy can damage genomic DNA, which, in turn, may activate NAS pathways. Activation of NAS pathways can lead to cytokine secretion and CD8^+^ T cell infiltration, enhancing the impact of radiotherapy and chemotherapy (13, 14). Moreover, activating nucleic acid sensors like STING stimulates T cell proliferation and may also induce vascular collapse, contributing to tumor cell death and apoptosis and potentially increasing the release of tumor-associated antigens (15–17). The diverse functions of the cGAS-STING pathway in modulating the immune microenvironment hold significant promise in the context of immunotherapy. Studies have shown that STING agonists can overcome resistance to anti-PD-1 agents in mouse tumor models (18). All the evidence thus reinforces the critical role of NAS pathways in cancer therapy (19–22). However, a comprehensive analysis of the relationship between NAS pathways and SCLC remains to be conducted.

Consequently, this study intends to elucidate the specific roles of NAS pathways in SCLC and to create a new risk model founded on genes associated with NAS pathways, aiming to predict the prognosis and response to treatment in patients with SCLC.

## Materials and methods

### Data collection

The nucleic acid-sensing (NAS) pathways-related genes were acquired from GSEA gene sets, path cards (https://pathcards.genecards.org/), and published articles. By eliminating duplicated genes, 371 NAS-related genes were included in this study. Three independent SCLC cohorts were enrolled in this study, including 79 SCLC patients collected from cBioPortal, 21 samples from GSE30219, and our cohort including 14 SCLC patients from Sichuan Provincial People’s Hospital, University of Electronic Science and Technology of China. This study was approved by the Ethics Committee of Sichuan Province People’s Hospital, School of Medicine, University of Electronic Science and Technology of China (No.20240122). The clinicopathological characteristics of the 14 patients were provided in **Supplementary Table 1**. The raw bulk transcriptome counts data, normalized and log2 converted RNA-sequencing (RNA-seq) profiles FPKM and normalized RMA of SCLC patients, and normal samples were acquired from cBioPortal (https://www.cBioPortal.org/) and GEO (https://www.ncbi.nlm.nih.gov/geo/). Normal samples and SCLC samples without complete clinical information were excluded from this study. The “GeoTcgaData” R package was applied to convert ensemble IDs to gene symbols.

### Expression distributions and variation levels of nucleic acid-sensing risk genes in SCLC

To identify the differentially expressed genes (DEGs) between SCLC and normal samples, the “limma” package was used with the criterion of the adjust. *P* < 0.05 and |log2FC| > 1.0.

### Consensus clustering analysis of NAS-related genes

Based on the expression profiles of 371 NAS-related genes, unsupervised classes of SCLC datasets were estimated with the consensus clustering method using the ‘Consensus Cluster-Plus 1.60.0’ package in R, and two clusters were obtained. Principal Component Analysis (PCA) was used to verify the clusters based on the expression profiles of the above genes.

### Relationship between NAS patterns with the prognosis of SCLC patients

To explore the differences in overall survival (OS) of the two clusters identified by consensus clustering, we conducted a Kaplan–Meier analysis generated by the ‘Survival’ and ‘Survminer’ packages of R.

### Construction of the NAS-related patterns

The least absolute shrinkage and selection operator (LASSO) method was used to screen out the genes that make a difference to the OS of SCLC patients, and a total of 12 genes were regarded as candidate genes. Based on the 12 genes, multivariate Cox regression was utilized to construct the final signature. Ultimately, the risk score of SCLC patients was calculated as: Risk score= ∑Coefficient of(i)× Expression of the gene(i). The patients could be divided into high-risk and low-risk groups according to the median risk score.

### Fluorescence-based multiplex immunohistochemistry staining

The tissue slides of SCLC patients (n = 14) were stained with multiplex fluorescence using PanoPANEL mIHC Kits (#0004100100). The slides were dewaxed by xylene and rehydrated by ethanol, and then the slides were heated in a microwave with antigen retrieval. The slides were incubated with primary antibody overnight at 4°C for specific antigen binding including anti-IRF1 (#8478, CST, USA), anti-CD45 (#13917, CST, USA), anti-CD68 (#97778. CST, USA), anti-CD8 (#85336, CST, USA), anti-panCK (#4545, CST, USA), anti-FoxP3 (#12653, CST, USA). The corresponding secondary antibodies should be pipetted onto the slides, and incubated at room temperature for 1 hour. DAPI was used to stain cell nuclei and at last the slides were sealed. The staining was scored by image J software based on the intensity and degree of staining. The degree of staining was compared using the Wilcoxon rank-sum test.

### Relationship between risk score and clinical characteristics

To evaluate the clinical significance of the risk score, we compared the associations between risk scores and clinicopathological features. The characteristics of the patients included survival status and subtypes (SCLC-A, SCLC-N, SCLC-P, and SCLC-Y). We used the “clusterProfiler” R package to identify differences in biological function between the high-risk and low-risk groups. Univariate and multivariate Cox regression analyses were performed to determine whether the risk model could be an independent prognostic factor for SCLC patients. We constructed a nomogram to predict the 1-, 2-, and 3-year OS of SCLC patients by the “RMS” R package.

### The analysis of the immune microenvironment

To explore the landscape of the immune microenvironment, the CIBERSORT algorithm was used to measure the infiltration of immune cells.

### Prediction of the response to immunotherapy and chemotherapy

The TIDE (Tumor Immune Dysfunction and Exclusion) score was calculated in TIDE (http://tide.dfci.harvard.edu/), which is usually used to predict the response to immunotherapy for cancer patients. The ‘oncoPredict’ package was used to predict the sensitivity of SCLC patients at different risks to chemotherapy drugs.

### Statistical analysis

Statistical analyses were performed using GraphPad Prism (v.9.0) (for experimental data), and R (v.4.2.1), and RStudio (v.3.5.3) (for sequencing data and matched clinical variables). Comparisons between groups were conducted using χ2 tests for categorical variables. Student’s t test was used for continuous variables. All the tests were two tailed, and *P* < 0.05 was considered statistically significant.

## Results

### NAS-related genes cluster SCLC patients into distinct subtypes

**Figure 1:**
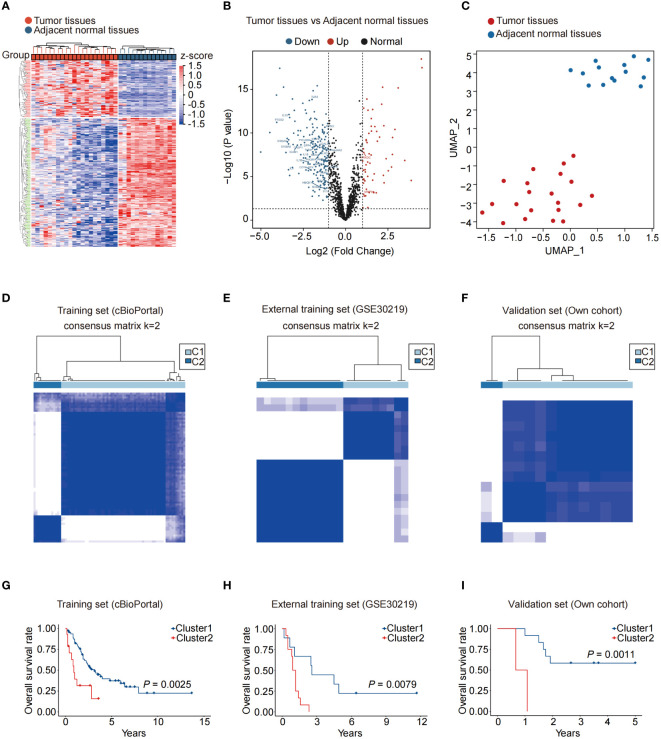
NAS-related genes can effectively distinguish and predict the prognosis of small cell lung cancer patients. **(A)** Heatmap of the differentially expressed genes between normal and SCLC tissues. **(B)** Volcano map of differentially expressed NAS-related genes between normal and SCLC tissues. The red dots represented upregulated genes in SCLC tissues, and the blue dots represented downregulated genes in SCLC tissues. **(C)** UMAP of normal and SCLC tissues. **(D–F)** According to the expression of NAS related genes, the SCLC patients were divided into two clusters in the training set **(D)**, external training set **(E)**, and our own validation set **(F)** (k = 2). **(G–I)** Kaplan-Meier analysis of the two SCLC subtypes in the training set **(G)**, external training set **(H)**, and own validation set **(I)**. NAS, Nucleic acid-sensing; SCLC, Small cell lung cancer; UMAP, Uniform Manifold Approximation and Projection.

Before analysis, we adjusted the transcriptome data from public datasets and our dataset (**Supplementary Figure S1A**). The GSE30219 cohort yielded 271 differentially expressed genes (DEGs) compared to adjacent normal tissues (**Figure 1A**). Among the 271 DEGs, 164 genes are upregulated, and 107 genes are downregulated, including several NAS related genes, such as *TLR3, PTGS2, IL-6, PRKDC*, and *MMP9* (**Figure 1B**). The UMAP analysis revealed that NAS related genes performed well in distinguishing cancer tissues and adjacent normal tissues (**Figure 1C**). We then conducted consensus clustering analysis to identify novel subtypes of SCLC based on NAS related genes. It demonstrated that the SCLC patients could be well classified into two clusters when k = 2 (**Figures 1D–F**; **Supplementary Figures S1B–D**). The PCA analysis, used to validate the clusters with respect to the expression profiles of NAS-related genes, showed a precise classification of the two clusters (**Supplementary Figures S1E-G**). More importantly, a notable disparity was observed in the OS time between the two clusters. Patients in Cluster 1 exhibited a favorable prognosis, whereas those in Cluster 2 showed a poor prognosis (**Figure 1G**). Similar results were validated in two additional SCLC cohorts, including the GEO cohort and our own cohort (**Figures 1H, I**). Overall, SCLC patients can be effectively classified into two groups based on the expression of NAS-related genes and the patients in cluster 1 have a better prognosis than patients in cluster 2.

### Construction of a prognostic gene signature based on 7 NAS-related genes for SCLC patients

**Figure 2:**
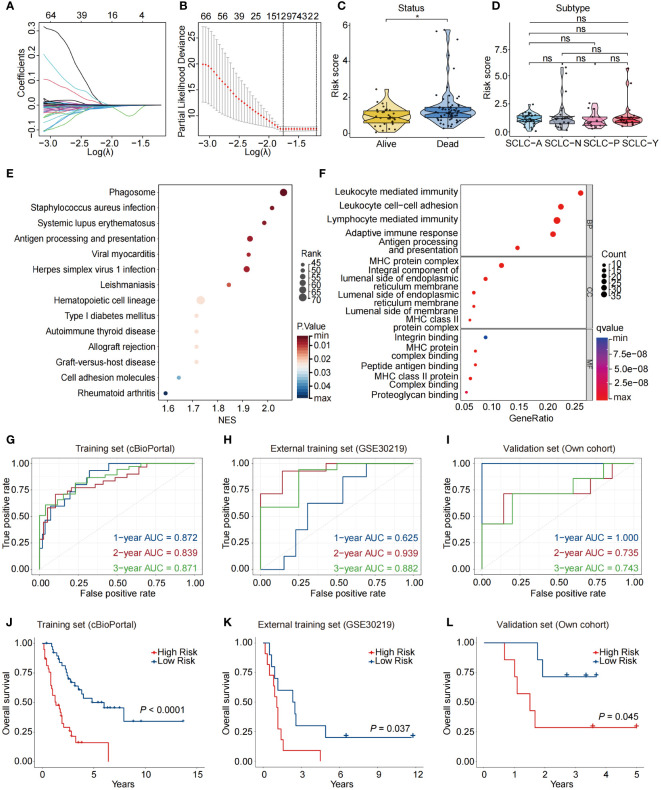
Construction of a prognostic model based on NAS related genes. **(A)** LASSO coefficient profiles of the twelve differentially expressed NAS related genes in SCLC patients. **(B)** The two dotted vertical lines are drawn at the optimal values by the minimum criteria (left) and 1−SE criteria (right). **(C)** Violin plots showing the correlation between risk score and survival status of SCLC patients. **(D)** Violin plots showing the correlation between risk score and SCLC subtypes. **(E, F)** KEGG **(E)** and GO **(F)** enrichment analysis based on the differentially expressed NAS genes between high-risk and low-risk groups. **(G–I)** Data are the area under the curve (AUC) of the risk scores in training set **(G)**, external training set **(H)**, and own validation set **(I)**. **(J–L)** Kaplan-Meier analysis of the prognosis of high-risk and low-risk groups in training set **(J)**, external training set **(K)**, and own validation set **(L)**. The *P* values are calculated by log-rank test for survival, unpaired, two-tailed Student’s *t* test, or one-way ANOVA. *P < 0.05. ns, non-significant; LASSO, Least Absolute Shrinkage and Selection Operator; GO, Gene Ontology; KEGG, Kyoto Encyclopedia of Genes and Genomes.

To construct a prognostic model based on a signature of NAS related genes, we conducted LASSO regression analysis and identified a total of 12 genes (**Figures 2A, B**). The multivariate Cox regression analysis demonstrated that there are 7 genes are related to the prognosis of patients, including *APOH*, *IL23A*, *IRF1*, *MAPKAPK2*, *POLR2E*, *TF*, and *UBA1*(**Supplementary Figures S2A–G**). Using the expression levels and coefficients of the 7 model genes, we constructed a prognostic gene signature (7NAS risk model) and calculated the risk score for each patient using the provided formula: Risk score = (-0.05773 * *APOH*) + (-0.01659 * *IL23A*) + (-0.01181 * *IRF1*) + (-0.01023 * *MAPKAPK2*) + (-0.01169 * *POLR2E*) + (-0.03609 * *TF*) + (-0.00676 * *UBA1*). Our results demonstrated that the risk score correlated with clinical characteristics such as alive or dead survival status but not with the subtypes of SCLC patients (**Figures 2C, D**). The low-risk groups significantly exhibit enrichment of multiple biological processes, particularly immune-related functions, such as adaptive immune response, antigen processing and presentation, and MHC protein complex binding (**Figures 2E, F**). We further analyzed the 7NAS risk model in predicting OS in both the training and validation sets (**Figures 2G–I**). Notably, a significant disparity was observed in the OS time between the high-risk and low-risk groups. Patients in the low-risk group demonstrated a tendency towards longer OS time in both the training and validation cohorts (*P* < 0.05) (**Figures 2J–L**). Collectively, we constructed a 7NAS risk model that could effectively predict the immune function status and OS of SCLC patients.

### The 7NAS risk score is an independent prognostic factor for SCLC patients

**Figure 3:**
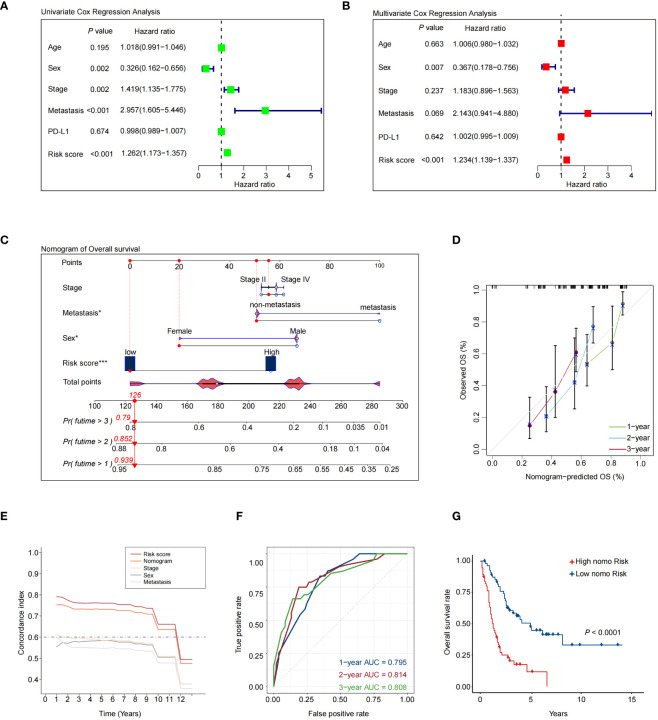
Validation of a nomogram that combines the 7NAS risk scores, sex, and stage to predict the overall survival of SCLC patients. **(A)** The univariate Cox regression analysis. **(B)** The multivariate Cox regression analysis. **(C)** The nomogram model was constructed to predict the prognosis of SCLC patients. **(D)** Regarding 1-, 2-, and 3-year survival of SCLC patients, the calibration curves showed that the predicted values were consistent with the observed values. **(E)** Concordance index analysis of nomogram. **(F)** ROC curves showed the performance of the nomogram in the Merged cohort. **(G)** The Kaplan-Meier analysis showed the overall survival between patients with low and high-risk scores. The *P* values are calculated by log-rank test for survival. ROC, Receiver Operating Characteristic curve. *P < 0.05; ***P < 0.001.

Next, we conducted univariate and multivariate Cox regression analyses to validate whether the 7NAS risk score could stand as an independent prognostic factor in SCLC. The univariate Cox regression analysis illustrated that the risk score constituted a risk factor in SCLC (HR = 1.262, 95% CI: 1.173-1.357, *P* < 0.001, **Figure 3A**). Following adjustment for confounding factors, the multivariate analysis also indicated that the risk score remained an independent prognostic factor (HR = 1.234, 95% CI: 1.139-1.337, *P* < 0.001, **Figure 3B**) for SCLC patients. A nomogram was developed to estimate the 1-, 2-, and 3-year OS, incorporating sex, tumor stage, metastasis and risk score (**Figure 3C**). Calibration curves demonstrated the predictive accuracy of this model for 1-, 2-, and 3-year survival rates (**Figure 3D**). It can be observed that the concordance index of nomogram is significantly better than clinical stage and metastasis, but slightly lower than the risk score of 7NAS model (**Figure 3E**). We assessed the area under the curve (AUC) values in a merged cohort which demonstrated the high accuracy in predicting 1-, 2-, and 3-year survival among SCLC patients (**Figure 3F**). The high-nomo-risk group commonly has a worse prognosis than the low-nomo-risk group (**Figure 3G**). Collectively, the 7NAS risk model represents an independent prognostic factor for patients with SCLC.

### The immune landscape related to the 7NAS risk scores in SCLC

**Figure 4:**
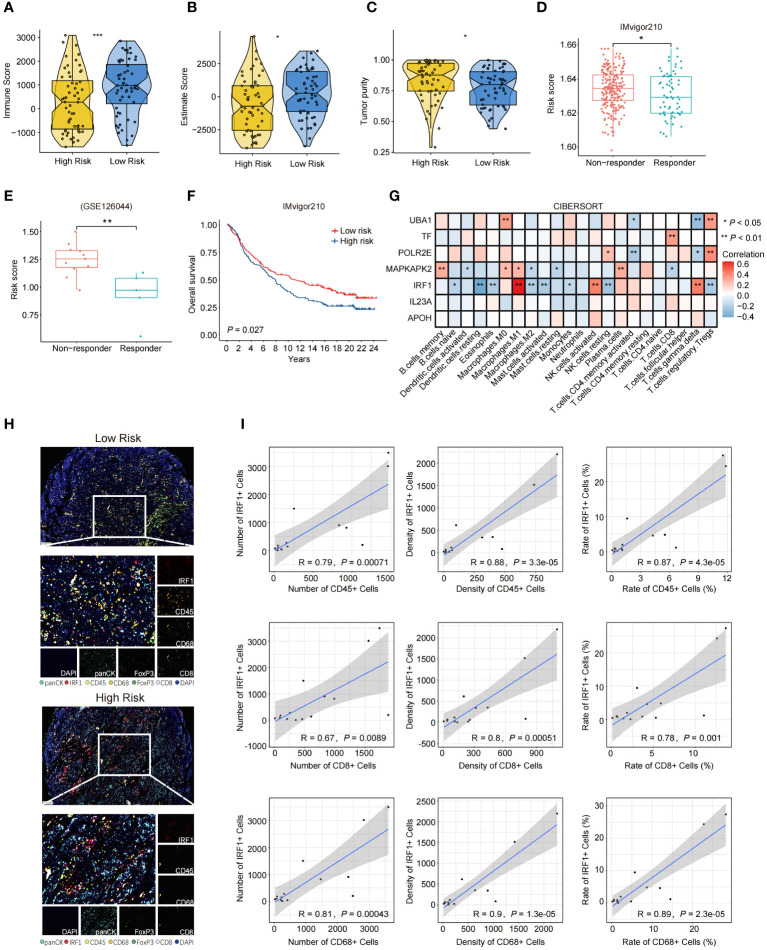
The immune landscape characterized based on the 7NAS risk model and *IRF1* gene. **(A–C)** The violin plot shows the immune score **(A)** Estimate Score **(B)**, and Tumor purity **(C)** of the low-risk group and the high-risk group. **(D, E)** The box plots present the response efficiency to immunotherapy of high-risk and low-risk groups in IMvigor210 **(D)** and GSE126044 **(E)** dataset. **(F)** Kaplan-Meier curve of overall survival for patients with high and low-risk groups in IMvigor210 dataset. **(G)** The heatmap shows the relationship between different model-related genes and the enrichment of immune cells. **(H)** Representative images of mIHC panel (panCK, CD45, CD8, CD68, FoxP3, and IRF1) in tumor tissues between high risk and low risk SCLC patients (Scale bar = 100 μm). **(I)** Correlation scatter plot showing the relationship between the expression of *IRF1* and the number, density, and rate of CD45^+^, CD8^+^, and CD68^+^ immune cells. The *P* values are calculated by unpaired, two-tailed Student’s *t* test. *P < 0.05; **P < 0.01; ***P < 0.001.

We analyzed the relationships between the risk scores and the immune characteristics of SCLC patients. The results revealed distinct immune microenvironments between the high-risk and low-risk groups. Specifically, SCLC patients in the low-risk group exhibited higher infiltration of immune cells in the merged dataset that combined the public dataset and our dataset (**Figures 4A–C**). The patients with lower risk scores were more sensitive to immunotherapy in IMvigor210 and GSE126044 datasets (**Figures 4D, E**), and the patients with lower risk scores tended to have better OS in IMvigor210 (**Figure 4F**). We further analyzed the correlation between the expression of the 7 NAS-related genes and the infiltration of intra-tumoral immune cells, separately. The results indicated that IRF1 was correlated with the infiltration of M1 macrophages, NK cells, and γδ T cells (**Figure 4G**). Further confirmation through mIHC revealed a positive correlation between high *IRF1* expression and the infiltration of CD45^+^ immune cells, CD8^+^ T lymphocytes, and CD68^+^ macrophages (**Figures 4H, I**). These results showed that the 7NAS risk score, especially the *IRF1* expression, has a positive relationship with the activated immune microenvironment.

### The effectiveness of the 7NAS risk score in predicting drug sensitivity of SCLC patients

**Figure 5:**
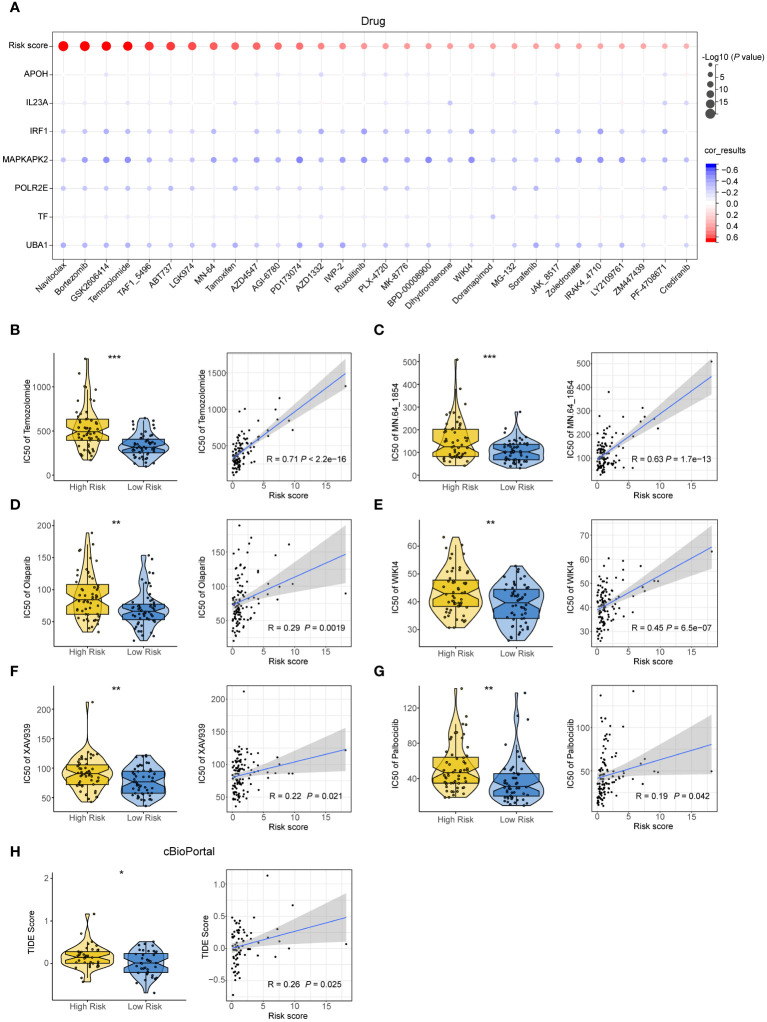
The effectiveness of the 7NAS risk model in predicting drug sensitivity. **(A)** Bar plots showing the relationship between IC50s of commonly used drugs and the expressions of the model genes. **(B–G)** The violin plot (left) and correlation scatter plot (right) show the IC50s between the high-risk group and low-risk group and the correlation of risk scores and IC50s of Temozolomide **(B)**, MN.64 1854 **(C)**, Olaparib **(D)**, WIKI4 **(E)**, XAV939 **(F)**, Palbociclib **(G)**. **(H)** The violin plot and correlation scatter plot show the TIDE score of the high-risk group and low-risk group in the cBioPortal cohorts. The *P* values are calculated by unpaired, two-tailed Student’s *t* test. *P < 0.05; **P < 0.01; ***P < 0.001.

To examine the predictive role of the 7NAS risk score in drug sensitivity of SCLC patients, we determined the half-maximal inhibitory concentration (IC50) value for each drug. The IC50 values of these chemotherapeutic agents exhibited a positive correlation with the risk score (**Figure 5A**), suggesting that patients with a low-risk score generally exhibited better responses to chemotherapy. Furthermore, in the case of Temozolomide (a second-line drug for SCLC patients) and PARP inhibitors, including MN.64_1864, Olaparib, WIKI4, XAV939, and Palbociclib, patients in the low-risk group exhibited greater sensitivity compared to those in the high-risk group (**Figures 5B–G**). Moreover, patients in the low-risk group decreased the TIDE score compared to the high-risk group from cBioPortal (**Figure 5H**). Conclusively, the 7NAS risk score plays a role in predicting patients who are sensitive to chemotherapy and immunotherapy.

## Discussion

SCLC is a high-grade malignant epithelial tumor and the lack of a specific molecular target limits the treatment approaches (1, 23, 24). In this study, we constructed a risk model based on the expression of 7 NAS-related genes and validated the prognostic and therapeutic efficacy prediction value of the model in different cohorts, including cBioPortal, GSE30219, and our cohort.

Despite emerging recognition of the subtypes of SCLCs based on high levels of ASCL1 (SCLC-A subtype), NEUROD1 (SCLC-N), POU2F3 (SCLC-P) or YAP1 (SCLC-Y), and other biomarkers including gene amplifications on 4q12 and CCNE1 amplification that could predict the overall survival of patients, the clinical approach to treatment is consistent irrespective (5, 25, 26). How the subtypes and other biomarkers influence therapeutic responsiveness and patterns of disease progression still needs to be further explored. Here, our study demonstrated that the 7NAS risk score could effectively predict the prognosis and immunotherapy response of patients. However, the high and low 7NAS risk scores have no significant variation among the four subtypes of patients with SCLCs.

SCLCs can be recognized by cytotoxic T cells, leading to durable benefit from immunotherapy (27, 28). While, this benefit has been seen in only a small minority of patients with metastatic SCLC (6, 8, 29). How to predict the immune responses of SCLC patients? Our study demonstrated that the patients with low 7NAS risk scores were mainly enriched in leukocyte-mediated immunity, adaptive immune response, antigen processing and presentation, and MHC protein complex. Moreover, the patients with lower 7NAS risk scores have a better response to immunotherapy and commonly have a long overall survival time compared to the patients high 7NAS risk scores underwent immunotherapy. IRF1 is the key transcription factor downstream of IFNγ which can be induced by STAT1 (30, 31). Here, in this study we demonstrated that IRF1 was most strongly correlated with the infiltration and functional roles of immune cells, suggesting it could serve as a potential target for enhancing the effectiveness of immunotherapy for SCLC patients.

In addition, we further explored the relationship between risk scores and the IC50 values of chemotherapeutics. The IC50s of most chemotherapeutics were positively correlated with the risk score, suggesting that patients in the low-risk group were more responsive to chemotherapy. Recent research has shown that a combination of Temozobmide and PARP inhibitors could improve the prognosis of SCLC (32, 33). Interestingly, our research suggested that patients in the low-risk group may have better responses to Temozobmide and PARP inhibitors, such as Olaparib.

Several limitations of our study must be acknowledged. Firstly, this was a retrospective cohort study with a relatively small number of patients, and the results need to be validated in future large prospective clinical trials. Secondly, no relevant information on immunotherapy was collected for all small cell lung cancer patients in this study. The predictive role of 7NAS risk scores on the effectiveness of immunotherapy and the prognosis of patient was based on the analysis of immunotherapy databases of bladder cancer patients. In future studies, more small cell lung cancer patients receiving immunotherapy will be collected to explore its effects. Last but not least, the treatment strategies for SCLC patients in multicenter cohorts are different. Although our final model exhibited robust and accurate predictive potential in patients who received different treatment options, to further ensure its predictive ability, the results need to be validated in more well-designed clinical trials with larger patient cohorts.

Overall, the 7NAS model that constructed by 7 NAS-related genes has an accurate predictive efficiency in identifying the SCLC patients who possess the activated immune microenvironment and long-time prognosis. The 7NAS model and nomo-risk-model combined 7NAS scores, sex and stages could help clinicians select patients who possess the long-time overall survival and could benefit from chemo or immunotherapy. The 7NAS model and nomo-risk-model could guide treatment decisions based on differing risks to provide the best treatment strategy for patients with SCLC.

## Data availability statement

The datasets presented in this study can be found in online repositories. Our own cohort are accessible with controlled access through the Chinese National Genomics Data Center under PRJCA024932 (https://ngdc.cncb.ac.cn/bioproject/browse/PRJCA024932).

## Ethics statement

The studies involving humans were approved by the Ethics Committee of Sichuan Province People’s Hospital, School of Medicine, University of Electronic Science and Technology of China. The studies were conducted in accordance with the local legislation and institutional requirements. The participants provided their written informed consent to participate in this study.

## Author contributions

QL: Conceptualization, Data curation, Formal analysis, Methodology, Validation, Visualization, Writing – original draft. ZL: Data curation, Formal analysis, Methodology, Validation, Visualization, Writing – original draft. NL: Data curation, Methodology, Validation, Writing – review & editing. JL: Data curation, Project administration, Supervision, Writing – original draft. HW: Conceptualization, Data curation, Formal analysis, Project administration, Supervision, Validation, Writing – original draft, Writing – review & editing. JC: Funding acquisition, Project administration, Resources, Supervision, Writing – original draft, Writing – review & editing.

## Supplementary material

Supplementary Figure 1
**(A)** The data showed the adjustment of the transcriptome data in the training set, external training set and validation set. **(B–D)** The cumulative distribution map of consensus clustering in training set, external training set and validation set. **(E–G)** The principal component analysis graph of the two SCLC subtypes in the training set, external training set and validation set.

Supplementary Figure 2 The prognostic value of the 7 filtered DNA sensor-related genes. **(A)** The Kaplan-Meier analysis showed the prognostic value of IL23A. **(B)** The Kaplan-Meier analysis showed the prognostic value of MAPKAPK2. **(C)** The Kaplan-Meier analysis showed the prognostic value of APOH. **(D)** The Kaplan-Meier analysis showed the prognostic value of TF. **(E)** The Kaplan-Meier analysis showed the prognostic value of POLR2E. **(F)** The Kaplan-Meier analysis showed the prognostic value of UBA1. **(G)** The Kaplan-Meier analysis showed the prognostic value of IRF1. The *P* values are calculated by log-rank test for survival.
