# Supplementary figures and images for: Nucleic acid-sensing-related gene signature in predicting prognosis and treatment efficiency of small cell lung cancer patients
Source: Front Oncol. 2024 Apr 12;14:1394286. doi: 10.3389/fonc.2024.1394286 (PMC11045993; doi:10.3389/fonc.2024.1394286)

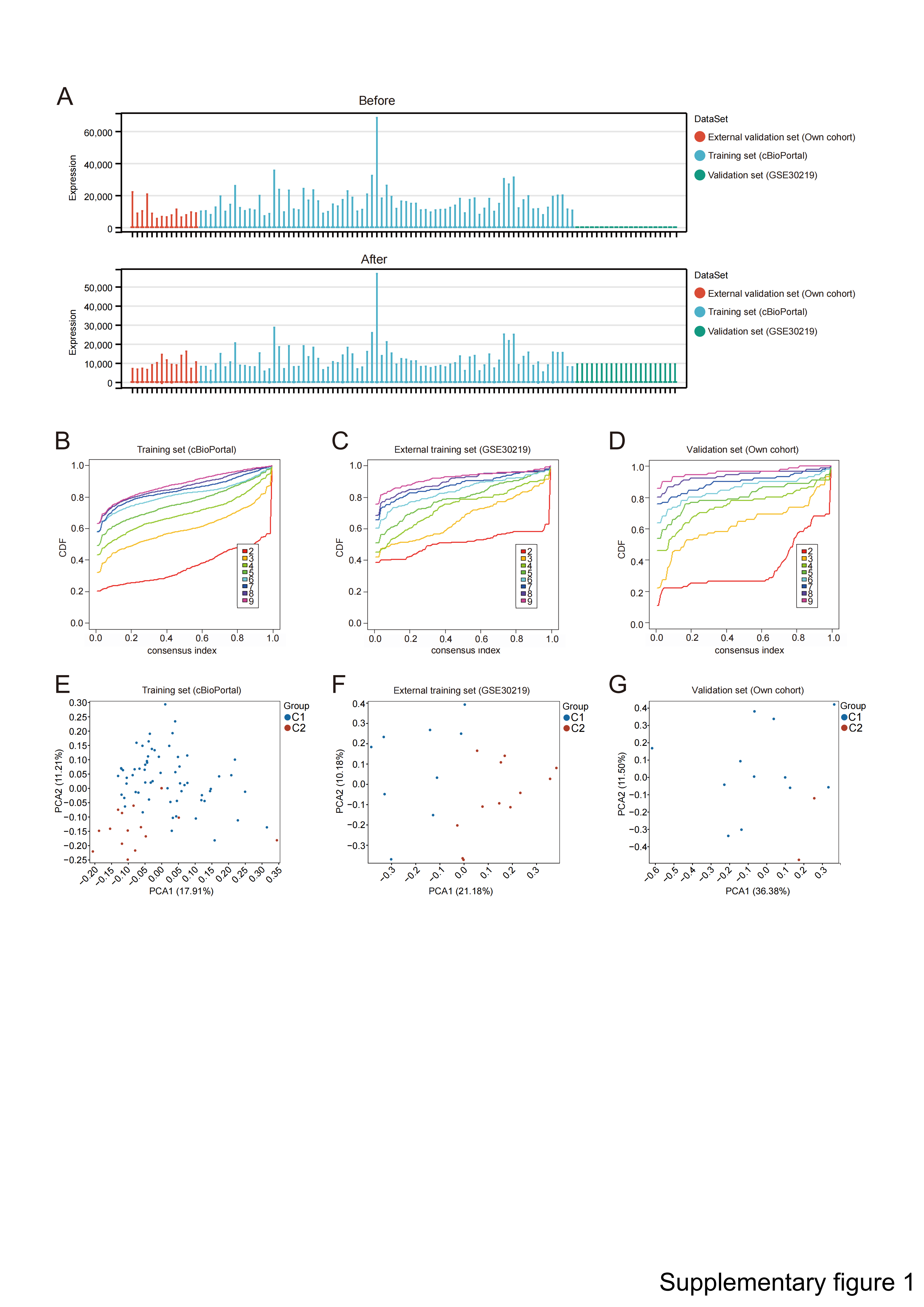

Supplement: Supplementary Figure 1 — (A) The data showed the adjustment of the transcriptome data in the training set, external training set and validation set. (B–D) The cumulative distribution map of consensus clustering in training set, external training set and validation set. (E–G) The principal component analysis graph of the two SCLC subtypes in the training set, external training set and validation set. [file Image_1.tif]

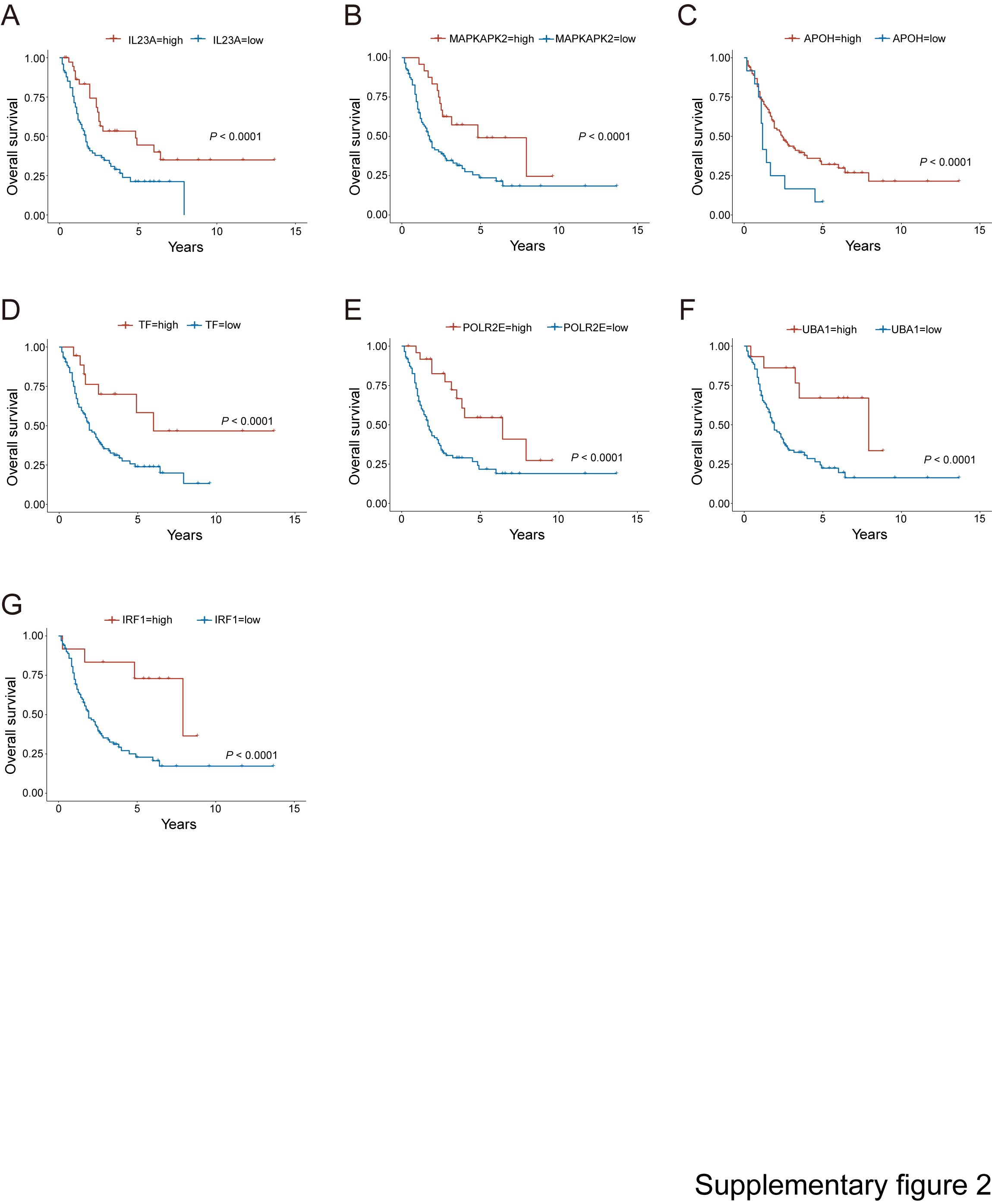

Supplement: Supplementary Figure 2 — The prognostic value of the 7 filtered DNA sensor-related genes. (A) The Kaplan-Meier analysis showed the prognostic value of IL23A. (B) The Kaplan-Meier analysis showed the prognostic value of MAPKAPK2. (C) The Kaplan-Meier analysis showed the prognostic value of APOH. (D) The Kaplan-Meier analysis showed the prognostic value of TF. (E) The Kaplan-Meier analysis showed the prognostic value of POLR2E. (F) The Kaplan-Meier analysis showed the prognostic value of UBA1. (G) The Kaplan-Meier analysis showed the prognostic value of IRF1. The P values are calculated by log-rank test for survival. [file Image_2.tif]
