# Supplementary material for: Nucleic acid-sensing-related gene signature in predicting prognosis and treatment efficiency of small cell lung cancer patients
Source: Front Oncol. 2024 Apr 12;14:1394286. doi: 10.3389/fonc.2024.1394286 (PMC11045993; doi:10.3389/fonc.2024.1394286)
Supplement: Supplementary file 3 [file Table_1.docx]

**Supplemental Table 1. The Clinicopathological Characteristics of SCLC Patients of Our Own Cohort.**

| **Sample** | **Gender** | **Age (years)** | **T status** | **N status** | **M status** | **Stage** |
| --- | --- | --- | --- | --- | --- | --- |
| 1 | Male | 55 | T2 | N2 | M0 | III |
| 2 | Male | 71 | T3 | N1 | M0 | III |
| 3 | Male | 54 | T3 | N2 | M1 | IV |
| 4 | Male | 53 | T2 | N3 | M0 | III |
| 5 | Male | 71 | T1 | N0 | M0 | I |
| 6 | Female | 59 | T2 | N3 | M0 | III |
| 7 | Male | 75 | T3 | N2 | M0 | III |
| 8 | Male | 79 | T1 | N0 | M0 | I |
| 9 | Male | 74 | T2 | N0 | M0 | I |
| 10 | Female | 59 | T2 | N2 | M0 | III |
| 11 | Male | 49 | T1 | N0 | Mx | I |
| 12 | Male | 77 | T3 | N1 | M0 | III |
| 13 | Male | 58 | T2 | N1 | M0 | III |
| 14 | Male | 67 | T3 | N0 | M0 | III |
